# On a Case of Cancerous Cachexia

**Published:** 1844-09-21

**Authors:** 


ON A CASE OF CANCEROUS CACHEXIA.

Catharine Guillot, ætat 49, was received in the wards of Professor Gerdy, at the Charité, and died there in the last stage of cancerous cachexia, having presented during her life-time a series of very remarkable accidents. Thus, in 1832, after an accouchment, abscess occurred in the right breast; in 1835, without any known cause, phlegmon developed itself in the same, and yielded only after two months' treatment; in 1841, amputation of this organ, on account of a cancer produced by pressure; in 1842, extirpation of a small cancer, which had developed itself in the neighborhood of the cicatrix, and of several axillary glands; a relapse, three months after; in 1843, intense lancinating pains in the right arm, which became swoln and fusiform, the bone being flexible, and, on pressure, crepitating, as if the seat of an osseous cyst; pain and numbness in all the extremities; soon after, development of a lancinating tumour, on the right side of the sternum, on a level with the third rib; characteristic tint; constipation. On the *post-mortem* examination, the body of the humerous was found to be re-placed by a medullary carcinoma, which had destroyed the bone from without inwards: the tumour on the sternum was of the same nature; it penetrated through that bone, into the interior of the chest, and adhered to the lungs. (The parts affected were, after removal, presented before the Academy of Medicine, sitting of the 9th July, by Professor Gerdy.)—*Lon. Medical Times.*

